# Supplementary figures and images for: Avian biodiversity in central California vineyards
Source: PeerJ. 2025 Aug 19;13:e19904. doi: 10.7717/peerj.19904 (PMC12372798; doi:10.7717/peerj.19904)

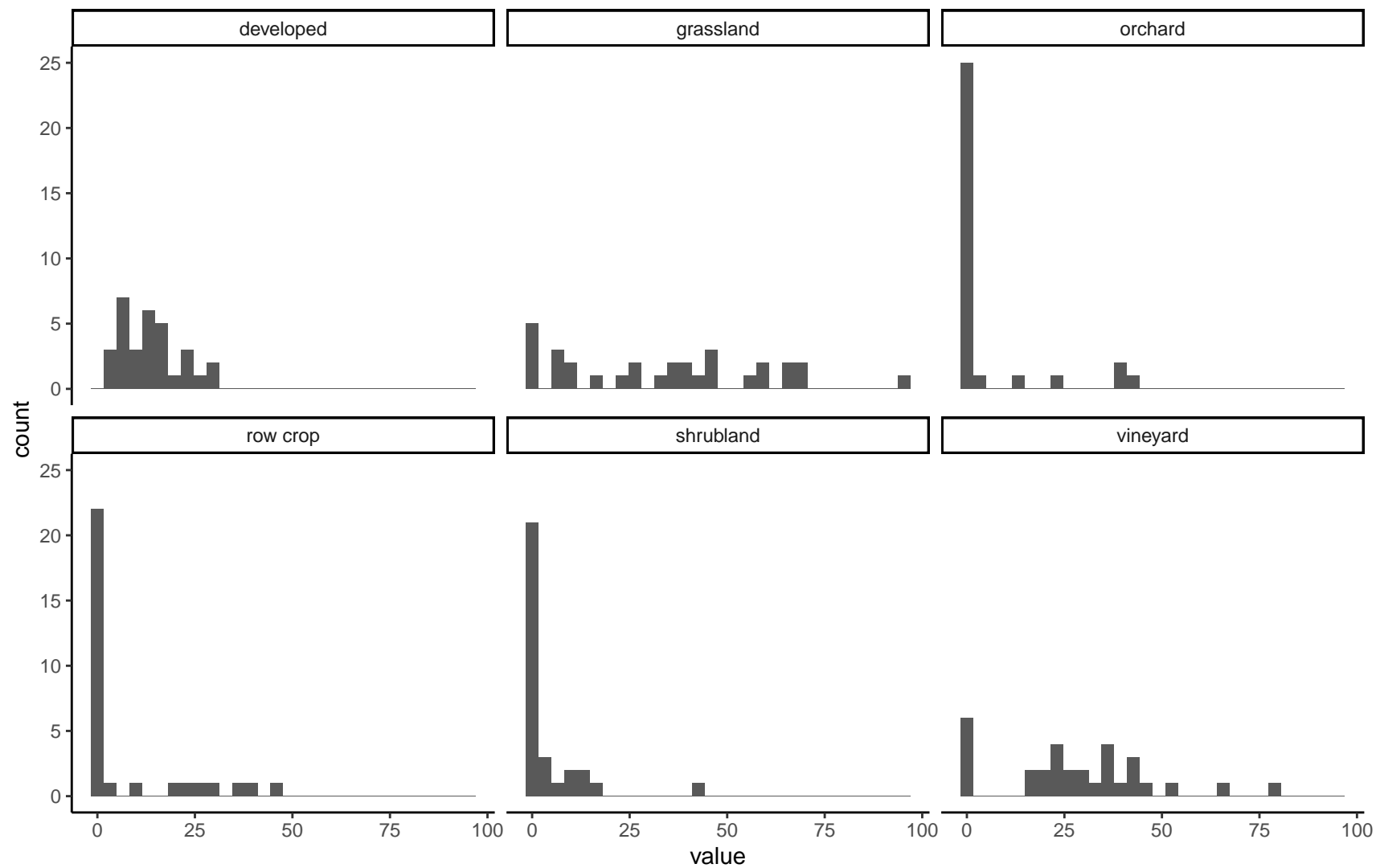

Supplement: Supplemental Information 1 [file peerj-13-19904-s001.pdf]

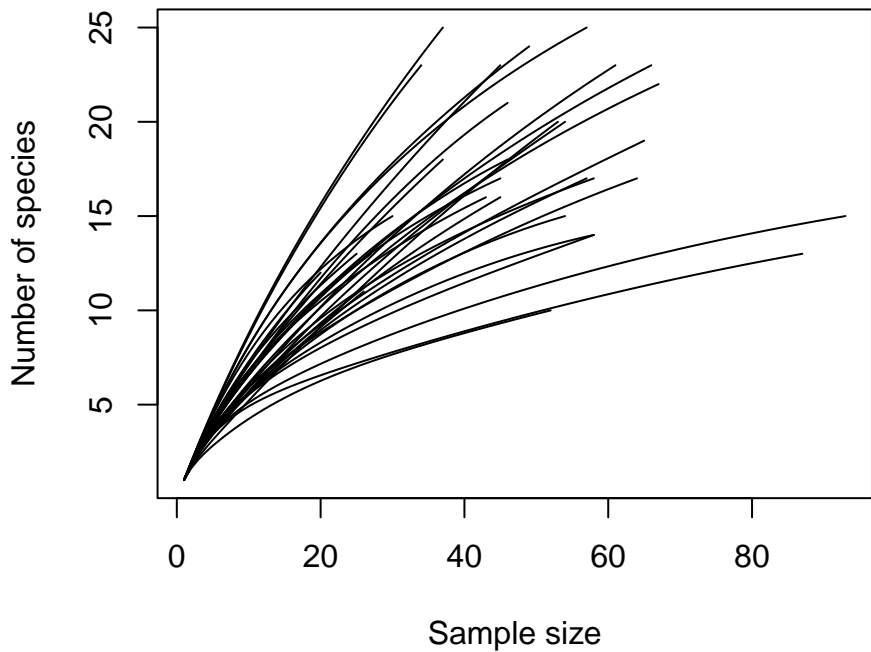

Supplement: Supplemental Information 2 [file peerj-13-19904-s002.pdf]
