# Supplementary material for: Avian biodiversity in central California vineyards
Source: PeerJ. 2025 Aug 19;13:e19904. doi: 10.7717/peerj.19904 (PMC12372798; doi:10.7717/peerj.19904)
Supplement: Supplemental Information 3 [file peerj-13-19904-s003.docx]

**Table S1. Detection probability for each species within a 100 and 150 radius.**

| **Species** | **Distance** | **Detection**  **probability** | **Species** | **Distance** | **Detection**  **probability** |
| --- | --- | --- | --- | --- | --- |
| Acorn woodpecker  *Melanerpes formicivorus* | 150 | 0.477 | European starling  *Sturnus vulgaris* | 150 | 0.435 |
|  | 100 | 0.404 |  | 100 | 0.432 |
| American crow  *Corvus brachyrhynchos* | 150 | 0.301 | Lesser goldfinch  *Spinus psaltria* | 150 | 0.392 |
|  | 100 | 0.235 |  | 100 | 0.363 |
| American robin  *Turdus migratorius* | 150 | 0.316 | Mourning dove  *Zenaida macroura* | 150 | 0.291 |
|  | 100 | 0.258 |  | 100 | 0.239 |
| Black phoebe  *Sayornis nigricans* | 150 | 0.342 | Nuttall’s woodpecker  *Dryobates nuttallii* | 150 | 0.166 |
|  | 100 | 0.321 |  | 100 | 0.166 |
| Brewer’s blackbird  *Euphagus cyanocephalus* | 150 | 0.252 | Oak titmouse  *Baeolophus inornatus* | 150 | 0.291 |
|  | 100 | 0.211 |  | 100 | 0.270 |
| Bushtit  *Psaltriparus minimus* | 100 | 0.291 | Red-shouldered hawk  *Buteo lineatus* | 100 | 0.193 |
|  | 150 | 0.291 |  | 150 | 0.193 |
| Cassin’s kingbird  *Tyranus vociferans* | 100 | 0.166 | Red-tailed hawk  *Buteo jamaicensis* | 150 | 0.240 |
|  | 150 | 0.166 |  | 100 | 0.239 |
| California towhee  *Melozone crissalis* | 150 | 0.426 | Song sparrow  *Melospiza melodia* | 150 | 0.482 |
|  | 100 | 0.414 |  | 100 | 0.385 |
| California quail  *Callipepla californica* | 150 | 0.429 | Tree swallow  *Tachycineta bicolor* | 150 | 0.232 |
|  | 100 | 0.223 |  | 100 | 0.200 |
| California scrub jay  *Aphelocoma californica* | 100 | 0.330 | Western bluebird  *Sialia mexicana* | 150 | 0.106 |
|  | 150 | 0.328 |  | 100 | 0.074 |
| Cliff swallow  *Petrochelidon pyrrhonota* | 100 | 0.232 | Western kingbird  *Tyrannus verticalis* | 100 | 0.074 |
|  | 150 | 0.232 |  | 150 | 0.074 |
| Eurasian collared-dove  *Streptopelia decaocto* | 150 | 0.240 | Wrentit  *Chamaea fasciata* | 150 | 0.385 |
|  | 100 | 0.197 |  | 100 | 0.185 |
