# Supplementary material for: Avian biodiversity in central California vineyards
Source: PeerJ. 2025 Aug 19;13:e19904. doi: 10.7717/peerj.19904 (PMC12372798; doi:10.7717/peerj.19904)
Supplement: Supplemental Information 4 [file peerj-13-19904-s004.docx]

**Table S2. Species occupancy *post hoc* models.**

| **Species** | **variable** | **estimate** | **SE** | **z** | **p-value** |
| --- | --- | --- | --- | --- | --- |
| Acorn woodpecker  *Melanerpes formicivorus* | **Canopy cover** | **0.130** | **0.064** | **2.020** | **0.044** |
|  | **Row crop cover** | **-0.179** | **0.087** | **-2.060** | **0.040** |
|  | **Sound** | **-0.403** | **0.199** | **-2.020** | **0.043** |
| American crow  *Corvus brachyrhynchos* | Vineyard cover | 0.082 | 0.064 | 1.287 | 0.198 |
|  | Row crop cover | -0.056 | 0.051 | -1.108 | 0.268 |
| American robin  *Turdus migratorius* | **Canopy height variation** | **-0.985** | **0.549** | **-1.795** | **0.073** |
|  | Canopy cover | 0.497 | 0.304 | 1.636 | 0.102 |
| Black phoebe  *Sayornis nigricans* | Vineyard cover | -0.076 | 0.085 | -0.887 | 0.375 |
|  | Developed cover | 0.059 | 0.072 | 0.821 | 0.412 |
|  | Shrubland cover | -0.108 | 0.127 | -0.850 | 0.396 |
| Brewer’s blackbird  *Euphagus cyanocephalus* | Vineyard cover | 0.990 | 0.856 | 1.170 | 0.243 |
|  | Developed cover | 0.616 | 0.582 | 1.060 | 0.290 |
| Bushtit  *Psaltriparus minimus* | Shrubland cover | 0.023 | 0.076 | 0.306 | 0.760 |
|  | Sound | -0.246 | 0.188 | -1.307 | 0.191 |
|  | Canopy height variation | 0.526 | 0.461 | 1.140 | 0.254 |
| Cassin’s kingbird  *Tyranus vociferans* | Orchard cover | -0.289 | 0.354 | -0.816 | 0.414 |
|  | Canopy height variation | -11.101 | 10.864 | -1.022 | 0.307 |
|  | Dist. to surface water | -0.091 | 0.072 | -1.255 | 0.210 |
| California towhee  *Melozone crissalis* | Grassland cover | 0.004 | 0.022 | 0.168 | 0.866 |
|  | Canopy cover | 0.045 | 0.058 | 0.781 | 0.435 |
|  | Developed cover | 0.122 | 0.094 | 1.295 | 0.195 |
| California quail  *Callipepla californica* | Grassland cover | -0.084 | 0.054 | -1.560 | 0.119 |
|  | **Canopy cover** | **0.469** | **0.246** | **1.910** | **0.057** |
|  | **Dist. to surface water** | **0.014** | **0.007** | **1.910** | **0.056** |
|  | **Sound** | **0.808** | **0.403** | **2.000** | **0.045** |
| California scrub jay  *Aphelocoma californica* | Developed cover | 0.100 | 0.071 | 1.406 | 0.160 |
|  | Orchard cover | -0.053 | 0.036 | -1.453 | 0.146 |
| Cliff swallow  *Petrochelidon pyrrhonota* | **Vineyard cover** | **0.062** | **0.030** | **2.030** | **0.042** |
|  | **Sound** | **0.366** | **0.219** | **1.670** | **0.095** |
| Eurasian collared-dove  *Streptopelia decaocto* | Vineyard cover | -0.061 | 0.038 | -1.590 | 0.112 |
|  | Grassland cover | -0.042 | 0.027 | -1.520 | 0.130 |
| European starling  *Sturnus vulgaris* | **Orchard cover** | **-0.123** | **0.067** | **-1.838** | **0.066** |
|  | Sound | -0.117 | 0.123 | -0.953 | 0.340 |
|  | Row crop cover | -0.071 | 0.045 | -1.596 | 0.110 |
| Lesser goldfinch  *Spinus psaltria* | Sound | -0.160 | 0.133 | -1.210 | 0.226 |
|  | Canopy cover | -0.118 | 0.113 | -1.049 | 0.294 |
|  | Canopy height variation | 0.399 | 0.570 | 0.699 | 0.484 |
| Mourning dove  *Zenaida macroura* | Vineyard cover | 3.785 | 10.861 | 0.349 | 0.727 |
|  | Row crop cover | -3.683 | 10.979 | -0.335 | 0.737 |
|  | Orchard cover | 2.812 | 92.438 | 0.030 | 0.976 |
| Nuttall’s woodpecker  *Dryobates nuttallii* | Canopy cover | 7.298 | 9.980 | 0.731 | 0.465 |
|  | Dist. to surface water | -0.305 | 0.450 | -0.677 | 0.499 |
| Oak titmouse  *Baeolophus inornatus* | Developed cover | 0.106 | 0.104 | 1.021 | 0.307 |
|  | Row crop cover | -0.030 | 0.038 | -0.769 | 0.442 |
| Red-shouldered hawk  *Buteo lineatus* | Developed cover | -4.900 | 4.790 | -1.020 | 0.307 |
|  | Sound | 8.640 | 8.130 | 1.060 | 0.288 |
|  | Canopy cover | 4.820 | 4.560 | 1.060 | 0.291 |
|  | Canopy height variation | -15.690 | 15.250 | -1.030 | 0.304 |
| Red-tailed hawk  *Buteo jamaicensis* | Shrubland cover | 0.466 | 0.436 | 1.067 | 0.286 |
|  | Grassland cover | -0.014 | 0.019 | -0.722 | 0.470 |
| Song sparrow  *Melospiza melodia* | **Dist. to surface water** | **-0.008** | **0.004** | **-1.780** | **0.076** |
|  | Orchard cover | -0.053 | 0.041 | -1.290 | 0.199 |
| Tree swallow  *Tachycineta bicolor* | Vineyard cover | 2.410 | 2.690 | 0.893 | 0.372 |
|  | Canopy height variation | 6.320 | 6.800 | 0.929 | 0.353 |
| Wrentit  *Chamaea fasciata* | **Shrubland cover** | **0.215** | **0.118** | **1.830** | **0.068** |

## 
