# Supplementary material for: Avian biodiversity in central California vineyards
Source: PeerJ. 2025 Aug 19;13:e19904. doi: 10.7717/peerj.19904 (PMC12372798; doi:10.7717/peerj.19904)
Supplement: Supplemental Information 5 — Bold predictor variables denote those with 90% confidence intervals (90% CI’s) that do not overlap zero. [file peerj-13-19904-s005.docx]

## **Table S3. Coverage-standardized Shannon index and species richness models.** Bold predictor variables denote those with 90% confidence intervals (90% CIs) that do not overlap zero.

| **Model** | **Variables** | **SI AIC_c_** | **SR AIC_c_** |
| --- | --- | --- | --- |
| Structural | **Canopy cover** + SD canopy + dist. to surface water | 187.4 | 191.8 |
| Natural cover | grassland cover + **shrubland cover** | 190.7 | 195.4 |
| Anthropic cover | **Vineyard cover** + developed cover + row crop cover + orchard cover + sound | 195.7 | 200.2 |
| Post hoc | **Canopy cover** + shrubland cover + vineyard cover | 186.3 | 190.9 |
