# Supplementary material for: Avian biodiversity in central California vineyards
Source: PeerJ. 2025 Aug 19;13:e19904. doi: 10.7717/peerj.19904 (PMC12372798; doi:10.7717/peerj.19904)
Supplement: Supplemental Information 6 [file peerj-13-19904-s006.docx]

**Table S4. Coverage-standardized Shannon index *post hoc* linear model.**

| **Coefficients** | **Estimate** | **Std. Error** | **t** | **p** |
| --- | --- | --- | --- | --- |
| Intercept | 7.597 | 1.746 | 4.351 | < 0.001 |
| poly(canopy, 2)1 | 6.418 | 4.999 | 1.284 | 0.210 |
| **poly(canopy, 2)2** | **-10.046** | **4.592** | **-2.188** | **0.038** |
| Vineyard cover | -0.026 | 0.050 | -0.524 | 0.604 |
| Shrubland cover | 0.162 | 0.101 | 1.598 | 0.122 |
