# Supplementary material for: Avian biodiversity in central California vineyards
Source: PeerJ. 2025 Aug 19;13:e19904. doi: 10.7717/peerj.19904 (PMC12372798; doi:10.7717/peerj.19904)
Supplement: Supplemental Information 7 [file peerj-13-19904-s007.docx]

**Table S5. Coverage-standardized species richness *post hoc* linear model.**

| **Coefficients** | **Estimate** | **Std. Error** | **t** | **p** |
| --- | --- | --- | --- | --- |
| Intercept | 8.178 | 1.880 | 4.350 | < 0.001 |
| poly(canopy, 2)1 | 6.965 | 5.384 | 1.294 | 0.207 |
| **poly(canopy, 2)2** | **-10.786** | **4.946** | **-2.181** | **0.038** |
| Vineyard cover | -0.029 | 0.054 | -0.535 | 0.597 |
| Shrubland cover | 0.170 | 0.109 | 1.559 | 0.131 |
