# Supplementary material for: Avian biodiversity in central California vineyards
Source: PeerJ. 2025 Aug 19;13:e19904. doi: 10.7717/peerj.19904 (PMC12372798; doi:10.7717/peerj.19904)
Supplement: Supplemental Information 8 — Bold predictor variables denote those with 90% confidence intervals (90% CI’s) that do not overlap zero. [file peerj-13-19904-s008.docx]

**Table S6. Beta diversity models.** Bold predictor variables denote those with 90% confidence intervals (90% CIs) that do not overlap zero.

| **Model** | **Variables** |
| --- | --- |
| Structural | **Canopy cover** + SD canopy height + distance to surface water |
| Natural cover | Grassland cover + shrubland cover |
| Agricultural cover | **Vineyard cover** + developed cover + orchard + row crop cover + **sound** |
| Post hoc | **Canopy cover** + **sound** + vineyard cover |
