# Supplementary material for: Avian biodiversity in central California vineyards
Source: PeerJ. 2025 Aug 19;13:e19904. doi: 10.7717/peerj.19904 (PMC12372798; doi:10.7717/peerj.19904)
Supplement: Supplemental Information 9 [file peerj-13-19904-s009.docx]

**Table S7. Beta diversity *post hoc* PERMANOVA.**

| **Variables** | **DF** | **Sum of Squares** | **R^2^** | **F** | **p** |
| --- | --- | --- | --- | --- | --- |
| Shrubland cover | 1 | 0.027 | 0.042 | 1.442 | 0.171 |
| **Canopy cover** | **1** | **0.041** | **0.065** | **2.202** | **0.016** |
| Vineyard cover | 1 | 0.016 | 0.026 | 0.889 | 0.578 |
| **Sound** | **1** | **0.031** | **0.050** | **1.697** | **0.062** |
| Residual | 26 | 0.481 | 0.764 |  |  |
| Total | 30 | 0.629 | 1.000 |  |  |
