# Supplementary material for: Avian biodiversity in central California vineyards
Source: PeerJ. 2025 Aug 19;13:e19904. doi: 10.7717/peerj.19904 (PMC12372798; doi:10.7717/peerj.19904)
Supplement: Supplemental Information 10 [file peerj-13-19904-s010.docx]

**Table S8. Four-letter code for species names.**

| **Species** | **Code** | **Species** | **Code** |
| --- | --- | --- | --- |
| Acorn woodpecker | ACWO | House wren | HOWR |
| American crow | AMCR | Lark sparrow | LASP |
| American robin | AMRO | Lesser goldfinch | LEGO |
| Anna’s hummingbird | ANHU | Mourning dove | MODO |
| Bewick’s wren | BEWR | Northern mockingbird | NOMO |
| Black phoebe | BLPH | Nuttall’s woodpecker | NUWO |
| Brewer’s blackbird | BRBL | Oak titmouse | OATI |
| Bushtit | BUSH | Red-shouldered hawk | RSHA |
| Cassin’s kingbird | CAKI | Red-tailed hawk | RTHA |
| California towhee | CALT | Red-winged blackbird | RWBL |
| California quail | CAQU | Song sparrow | SOSP |
| California scrub jay | CASJ | Spotted towhee | SPTO |
| Cliff swallow | CLSW | Turkey vulture | TUVU |
| Common yellowthroat | COYE | Western bluebird | WEBL |
| Eurasian-collared dove | EUCD | Western flycatcher | WEFL |
| European starling | EUST | Western kingbird | WEKI |
| House finch | HOFI | Wrentit | WREN |
