# Supplementary material for: Avian biodiversity in central California vineyards
Source: PeerJ. 2025 Aug 19;13:e19904. doi: 10.7717/peerj.19904 (PMC12372798; doi:10.7717/peerj.19904)
Supplement: Supplemental Information 11 — Bold predictor variables denote those with 90% confidence intervals (90% CI’s) that do not overlap zero. [file peerj-13-19904-s011.docx]

**Table S9. Functional evenness models.** Bold predictor variables denote those with 90% confidence intervals (90% CIs) that do not overlap zero.

| **Model** | **Variables** | **AIC_c_** |
| --- | --- | --- |
| Structural | Canopy cover + SD canopy + dist. to surface water | 283.4 |
| Natural cover | grassland cover + shrubland cover | 293.0 |
| Anthropic cover | **Vineyard cover** + developed cover + orchard cover + row crop cover + sound | 287.2 |
| Post hoc | **Vineyard cover** **+ dist. To surface water + canopy cover** | 280.6 |
