# Supplementary material for: Avian biodiversity in central California vineyards
Source: PeerJ. 2025 Aug 19;13:e19904. doi: 10.7717/peerj.19904 (PMC12372798; doi:10.7717/peerj.19904)
Supplement: Supplemental Information 12 [file peerj-13-19904-s012.docx]

**Table S10. Functional evenness *post hoc* linear model results.**

| **Coefficients** | **Estimate** | **Std. Error** | **t value** | **p** |
| --- | --- | --- | --- | --- |
| (Intercept) | 69.811 | 7.879 | 8.861 | 2.47e-9 |
| **poly(canopy, 2)1** | **48.324** | **22.916** | **2.109** | **0.074** |
| **poly(canopy, 2)2** | **-38.132** | **20.515** | **-1.859** | **0.045** |
| **Dist. to surface water** | **0.062** | **0.029** | **2.110** | **0.045** |
| **Vineyard cover** | **-0.0383** | **0.226** | **-1.738** | **0.094** |
