# Supplementary material for: Avian biodiversity in central California vineyards
Source: PeerJ. 2025 Aug 19;13:e19904. doi: 10.7717/peerj.19904 (PMC12372798; doi:10.7717/peerj.19904)
Supplement: Supplemental Information 13 — Bold predictor variables denote those with 90% confidence intervals (90% CI’s) that do not overlap zero. [file peerj-13-19904-s013.docx]

**Table S11. Functional dispersion models.** Bold predictor variables denote those with 90% confidence intervals (90% CIs) that do not overlap zero.

| **Model** | **Variables** | **AIC_c_** |
| --- | --- | --- |
| Structural | **Canopy cover** + SD canopy + dist. to surface water | 284.7 |
| Natural cover | **grassland cover** + shrubland cover | 284.9 |
| Anthropic cover | Vineyard cover + developed cover + orchard cover + **row crop cover** + sound | 284.7 |
| Post hoc | **Canopy cover** + **row crop cover** + vineyard cover | 278.2 |
