# Supplementary material for: Avian biodiversity in central California vineyards
Source: PeerJ. 2025 Aug 19;13:e19904. doi: 10.7717/peerj.19904 (PMC12372798; doi:10.7717/peerj.19904)
Supplement: Supplemental Information 14 [file peerj-13-19904-s014.docx]

**Table S12. Functional dispersion *post hoc* linear model.**

| **Coefficients** | **Estimate** | **Std. Error** | **t value** | **p** |
| --- | --- | --- | --- | --- |
| (Intercept) | 25.895 | 9.306 | 2.783 | 0.010 |
| Grassland cover | 0.025 | 0.173 | 0.146 | 0.885 |
| **Row crop cover** | **0.689** | **0.312** | **2.209** | **0.036** |
| **Canopy cover** | **0.713** | **0.302** | **2.360** | **0.026** |
