# Supplementary material for: Avian biodiversity in central California vineyards
Source: PeerJ. 2025 Aug 19;13:e19904. doi: 10.7717/peerj.19904 (PMC12372798; doi:10.7717/peerj.19904)
Supplement: Supplemental Information 15 — Bold predictor variables denote those with 90% confidence intervals (90% CI’s) that do not overlap zero. [file peerj-13-19904-s015.docx]

**Table S13. Functional richness models.** Bold predictor variables denote those with 90% confidence intervals (90% CIs) that do not overlap zero.

| **Model** | **Variables** | **AIC_c_** |
| --- | --- | --- |
| Structural | **Canopy cover** + SD canopy + dist. to surface water | 285.3 |
| Natural cover | grassland cover + shrubland cover | 298.7 |
| Anthropic cover | Vineyard cover + developed cover + orchard cover + row crop cover + **sound** | 296.5 |
| Post hoc | **Canopy cover** + **dist. to surface water** + sound | 285.0 |
