# Supplementary material for: Avian biodiversity in central California vineyards
Source: PeerJ. 2025 Aug 19;13:e19904. doi: 10.7717/peerj.19904 (PMC12372798; doi:10.7717/peerj.19904)
Supplement: Supplemental Information 16 [file peerj-13-19904-s016.docx]

**Table S14. Functional richness *post hoc* linear model.**

| **Coefficients** | **Estimate** | **Std. Error** | **t value** | **p** |
| --- | --- | --- | --- | --- |
| (Intercept) | 65.582 | 58.562 | 1.120 | 0.273 |
| **Canopy cover** | **1.078** | **0.385** | **2.798** | **0.009** |
| **Distance to surface water** | **-0.057** | **0.033** | **-1.728** | **0.095** |
| Sound | -0.688 | 0.911 | -0.755 | 0.457 |
